# Difficulty in Attention Switching and Its Neural Basis in Problematic Smartphone Use

**DOI:** 10.3390/brainsci15101100

**Published:** 2025-10-13

**Authors:** Nanase Kobayashi, Daisuke Jitoku, Toshitaka Hamamura, Masaru Honjo, Yusei Yamaguchi, Masaaki Shimizu, Shunsuke Takagi, Junya Fujino, Genichi Sugihara, Hidehiko Takahashi

**Affiliations:** 1 Department of Psychiatry, Institute Science Tokyo, Tokyo 113-8510, Japan; nanase.psyc@tmd.ac.jp (N.K.); jitopsyc@tmd.ac.jp (D.J.); yamay.psyc@tmd.ac.jp (Y.Y.); ma200036z@gmail.com (M.S.); takagi.s.659d@m.isct.ac.jp (S.T.); xjf15psyc@tmd.ac.jp (J.F.); hidepsyc@tmd.ac.jp (H.T.); 2 Institute of Biomedical Engineering, Medical Engineering Laboratory, Department of Cyber Psychiatry, Institute Science Tokyo, Tokyo 113-8510, Japan; 3 National Center for Cognitive Behavior Therapy and Research, National Center of Neurology and Psychiatry, Tokyo 187-8553, Japan; toshitaka.hamamura@gmail.com; 4 KDDI Research, Inc., Saitama 356-8502, Japan; honjo@kddi-research.jp; 5 Institute of Biomedical Engineering Center for Brain Integration Research, Institute of Science Tokyo, Tokyo 113-8510, Japan

**Keywords:** problematic smartphone use, nucleus accumbens, attentional switching, nighttime screen time, reward system, magnetic resonance imaging

## Abstract

**Background**: Problematic smartphone use (PSU) involves excessive smartphone engagement that disrupts daily functioning and is linked to attentional control deficits and altered reward processing. The nucleus accumbens (NAcc), a key structure in the reward system, may contribute to difficulty disengaging from rewarding digital content. This study examined relationships between NAcc volume, attentional switching, and objectively measured nighttime screen time in individuals with PSU. **Methods**: Fifty-three participants (aged ≥ 13 years) from an outpatient internet dependency clinic completed psychological assessments, brain MRI, and smartphone logging. PSU was diagnosed by two psychiatrists. Attentional switching was measured via the Autism Spectrum Quotient subscale. Nighttime screen time (00:00–06:00) was recorded via smartphone. MRI-derived NAcc volumes were normalized to total gray matter volume. Correlations, multiple regression (controlling for ASD and ADHD), and mediation analyses were conducted. **Results**: Difficulty in attention switching correlated with larger right NAcc volume (r = 0.45, *p* = 0.012) and increased nighttime screen time (r = 0.44, *p* = 0.014). Right NAcc volume also correlated with nighttime screen time (r = 0.46, *p* = 0.012). Regression showed right NAcc volume predicted nighttime screen time (β = 0.33, *p* = 0.022), whereas attentional switching was not significant. Mediation was unsupported. Sensitivity analyses confirmed associations. **Conclusions**: Larger right NAcc volume independently predicts prolonged nighttime smartphone use and is associated with impaired attentional switching in PSU. Structural variations in reward-related regions may underlie difficulty disengaging from digital content. Integrating neurobiological, cognitive, and behavioral measures offers a framework for understanding PSU.

## 1. Introduction

Smartphone usage has become an integral part of modern life, supporting a wide range of activities such as communication, social networking, and entertainment [1,2]. However, excessive smartphone engagement has raised growing concerns about its potential adverse effects on mental health and daily functioning. Problematic smartphone use (PSU)—characterized by excessive or compulsive use that interferes with everyday life—has been increasingly recognized as a behavioral addiction analogous to Internet Gaming Disorder (IGD) [3]; however, PSU is not a formal DSM/ICD diagnosis and, in this study, is treated as a research construct. PSU is frequently associated with psychological distress, academic decline, and social withdrawal [2], with prevalence estimates ranging from 10% to 30% among adolescents and young adults worldwide.

Disrupted sleep is commonly reported among individuals with PSU and is often the result of prolonged nighttime smartphone use driven by compulsive behavior or deep engagement with digital content. Such patterns may reflect a reduced ability to shift attention away from rewarding stimuli at bedtime. Although deficits in attentional switching have been implicated in PSU [4], the specific association between attentional switching difficulties and objectively measured nighttime screen time has not yet been examined.

The nucleus accumbens (NAcc), a key component of the brain’s reward circuitry, has been implicated in the neurobiology of behavioral addictions. Neuroimaging studies have demonstrated that increased NAcc volume is associated with heightened impulsivity and reward sensitivity [5,6], linking structural variations in this region to compulsive reward-seeking behaviors. PSU shares these core features of dysregulated reward processing and compulsivity. While executive functions such as attentional switching are traditionally attributed primarily to the prefrontal cortex, emerging evidence suggests that the NAcc also contributes, particularly in adapting to dynamic reward contexts [7]. This raises the possibility that NAcc structure influences the ability to disengage from highly salient, rewarding stimuli—a hallmark of PSU.

Based on these considerations, this study examined whether PSU severity is related to attentional switching difficulties and structural variations in the NAcc. We hypothesized that individuals with higher PSU severity would exhibit greater difficulty with attentional switching and longer nighttime screen time, both of which are associated with larger NAcc volume. A key strength of this study lies in its multi-level design, integrating behavioral measures (objectively recorded nighttime screen time), cognitive assessments (attentional switching), and neurobiological data (NAcc volume) to provide a comprehensive understanding of the mechanisms underlying PSU.

## 2. Materials and Methods

### 2.1. Participants

The study enrolled individuals who sought treatment at an outpatient internet dependency clinic affiliated with a university hospital between September 2019 and June 2022. In this study, Problematic Smartphone Use (PSU) refers to a research-defined category rather than an official diagnostic entity in classification systems such as the ICD-11 or DSM-5. PSU was operationalized by adapting the DSM-5 diagnostic criteria for Internet Gaming Disorder (IGD), replacing the term “Internet game” with “smartphone usage.” Other psychiatric disorders were also assessed by board-certified psychiatrists. Diagnoses of autism spectrum disorder (ASD) and attention-deficit/hyperactivity disorder (ADHD) were made according to the DSM-5 diagnostic criteria, and these comorbidities were recorded as supplementary diagnostic information.

Inclusion criteria encompassed men and women aged 13 or older who provided informed consent for the installation of a logging application on their smartphones to monitor usage and for undergoing brain MRI scans. Exclusion criteria comprised individuals with severe mental instability, those facing physical conditions that hindered outpatient visits, individuals with implanted metal devices such as pacemakers or cochlear implants, and those who experienced claustrophobia. Participants encountering technical difficulties related to parental controls set on iOS devices for downloading the application were also excluded. Additionally, individuals who had their smartphones confiscated or damaged due to problematic behavior, as well as those unable to download logging applications due to insufficient storage capacity, were not included in the study. No participants had any history of head trauma, serious medical or surgical illness, or substance abuse. All participants were not current smokers.

Data collection for the participants involved a comprehensive assessment consisting of self-administered psychological scales, utilization of a smartphone logging application, and brain MRI scans. Based on previous studies, all participants completed the Smartphone Addiction Scale Short Version [8,9,10,11,12,13] to measure the degree of dependence on smartphones. This test measures the degree of involvement in online activities using a six-point Likert scale and classifies addictive behaviors: a total score of ≥31 as “severe addictive behaviors.” In addition to the primary measures, the Attention-Deficit/Hyperactivity Disorder Rating Scale (ADHD-RS) was administered to assess symptoms of inattention and hyperactivity–impulsivity [14,15]. Although the ADHD-RS scores were included in the descriptive statistics, they were not analyzed in the main statistical models for the present study.

This study was approved by the institutional review board of Tokyo Medical and Dental University Hospital (currently Institute of Science Tokyo) (M2020-073) and was conducted in accordance with the Code of Ethics of the World Medical Association. After explaining the entire study, written informed consent was obtained from all participants and their parents.

### 2.2. Attention Switching

Attention switching was specifically evaluated using the “Attention Switching” subscale of the Autism Spectrum Quotient (AQ) [16]. While task-based measures, such as the Wisconsin Card Sorting Test (WCST) [17], offer objective data at a single point in time, their results can be influenced by factors such as fatigue or recent sleep quality [18,19]. Although questionnaires are inherently subjective, they capture cognitive tendencies over more extended periods. The AQ, originally developed as a screening instrument for autism spectrum disorder (ASD), features a distinct “Attention Switching” subscale [16]. In the official English version, the relevant items include statements such as: “I find it easy to do more than one thing at once,” “I find it difficult to do something if there is an interruption,” and “I tend to get so strongly absorbed in one thing that I lose sight of other things.” These items help quantify how individuals perceive and manage shifting their attention to different tasks or stimuli.

### 2.3. Nighttime Screen Time

We employed U-Logger, a smartphone logging application developed by KDDI Corporation and KDDI Research, Inc. (Saitama, Japan), to capture log data. Relying solely on self-reported data in PSU research may not provide a complete picture [20]. Objective measures, such as smartphone logs, offer a more detailed understanding, particularly when gauging the genuine social dysfunctions observed in patients.

U-Logger operated discreetly in the background without disrupting regular smartphone usage. Upon installation, the application assigned a random device ID for user identification. It recorded smartphone activities, including power status, program activations, and screen-on/off notifications. Importantly, U-Logger did not access personal content such as photos or messages. Collected data were securely stored on a dedicated log storage server. Participants were instructed to keep the application running continuously throughout the study period, avoid closing the application (for iOS users), ensure sufficient device memory and storage capacity, keep the device powered on, and grant necessary data collection permissions within the application. However, due to participants occasionally disabling the application, operating system restrictions (particularly on iOS), or device-specific technical issues, successful log acquisition was not always guaranteed. These factors could reduce the overall logging adherence. We utilized this data to assess the number of logged days and to measure nighttime screen time (from midnight to 6 a.m.). Screen time was determined based on screen-on/off events, as well as application launches. The log acquisition rate was calculated as the percentage of days during the initial two-week period following application installation when logs were successfully recorded.

### 2.4. Volumes of the Nucleus Accumbens

The MRI images were acquired on a 3-Tesla MRI system (Spectra, Siemens Healthcare, Tokyo, Japan) at Tokyo Medical and Dental University Hospital’s dental department. The high-resolution 3D T1-weighted images were obtained with the following parameters: repetition time (TR) = 1.8 ms; echo time (TE) = 2.42 ms; inversion time (TI) = 900 ms; flip angle (FA) = 9°; acquisition matrix = 256 × 256; slice thickness = 1 mm. In this study, we designated the volume of the nucleus accumbens (NAcc), a pivotal component of the brain’s reward system, as our region of interest, building upon established findings from prior research [5,6]. Structural MRI data were processed using the Computational Anatomy Toolbox (CAT12, version 12.8.2) [11], implemented in SPM12 (Statistical Parametric Mapping; Wellcome Centre for Human Neuroimaging, London, UK), running on MATLAB R2023a. Preprocessing included bias correction, segmentation into gray matter, white matter, and cerebrospinal fluid, and spatial normalization to MNI space using the DARTEL algorithm [8]. CAT12 performs these steps using advanced interpolation, local intensity correction, and adaptive tissue segmentation. Gray matter images were then used to extract the volume of the left and right NAcc using the Neuromorphometrics atlas included in CAT12. To account for individual differences in total brain size, NAcc volumes were normalized by dividing each value by the participant’s total gray matter volume. The resulting value was then scaled by multiplying it by the mean total gray matter volume across all participants.

### 2.5. Statistical Analysis

All statistical analyses were conducted using IBM SPSS Statistics (Version 28). The significance level for all tests was set at an alpha of *p* < 0.05, two-tailed.

Our primary analysis began with bivariate correlations to examine the relationships among the key variables: left and right NAcc volumes, attentional switching impairment, and nighttime screen time. Subsequently, a multiple regression analysis was performed to predict nighttime screen time. Based on the initial correlational results, only the right NAcc volume was selected as an independent variable alongside the attentional switching measure to ensure model parsimony and prevent multicollinearity. To control for potential confounding effects of neurodevelopmental conditions, diagnoses of ASD and ADHD were included as covariates in the regression model.

To explore potential indirect relationships, we conducted a mediation analysis using the PROCESS macro for SPSS (Model 4), with 5000 bootstrap resamples. We tested a hypothesized model in which the relationship between right NAcc volume (predictor, X) and nighttime screen time (outcome, Y) was mediated by attentional switching impairment (mediator, M). An indirect effect was considered statistically significant if its 95% bootstrapped confidence interval (CI) did not include zero.

Finally, a sensitivity analysis was performed to confirm the robustness of our findings. We calculated Spearman’s rank-order correlations among right NAcc volume, attentional switching impairment, and nighttime screen time within a subsample of 28 participants selected for high data-logging adherence (log acquisition rate > 35.7%, the sample median). This non-parametric analysis assessed whether the observed associations persisted in participants with the most complete datasets.

### 2.6. Generative AI Statement

Generative artificial intelligence (ChatGPT, OpenAI, GPT-4o and GPT-5, 2024–2025) was used to assist in translating the manuscript from Japanese to English. The translation was subsequently reviewed and edited by the authors to ensure accuracy, clarity, and alignment with the intended meaning. No AI tools were used for data analysis, study design, or interpretation of results.

## 3. Results

Participant demographic and clinical characteristics are summarized in Table 1. The sample included high rates of neurodevelopmental comorbidities; 33% of participants had a diagnosis of ASD, and 30% had a diagnosis of ADHD. As measured by the ADHD-RS, the ADHD presentation was predominantly inattentive.

**Figure 1:**
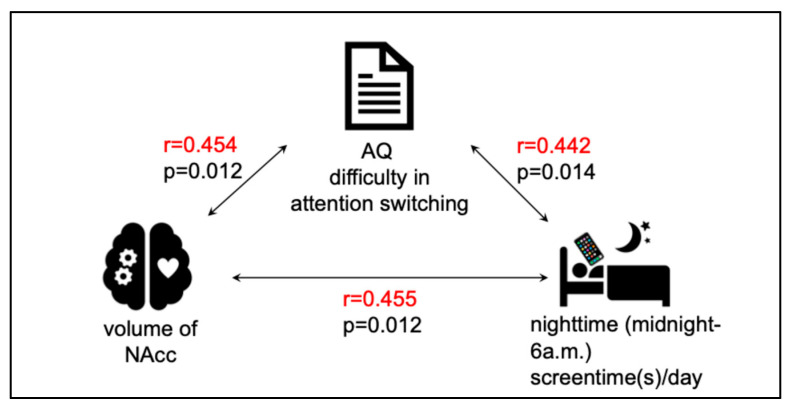
Significant correlations among NAcc volume, difficulty in attention switching, and nighttime screen time. The volume of the nucleus accumbens (NAcc), self-reported difficulty in attention switching (measured by the AQ subscale), and nighttime smartphone use (midnight–6 a.m.) were all significantly and positively correlated.

**Figure 2:**
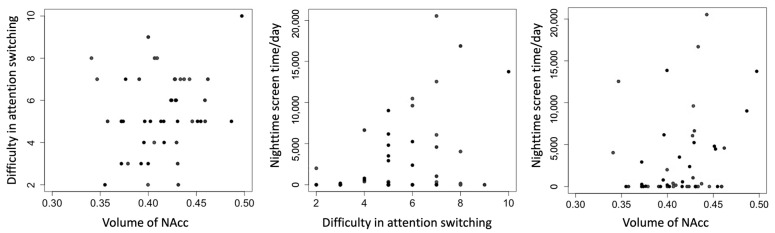
Scatterplots of difficulty in attention switching, nighttime screen time, and volume of NAcc. Each panel depicts a bivariate relationship among the three primary variables: right NAcc volume (arbitrary units, normalized to total gray matter volume), self-reported difficulty in attention switching (measured by the AQ subscale, range 0–10), and objectively logged nighttime smartphone use (seconds per day, midnight–6 a.m.). All data are plotted with raw values. Regression lines are not shown, as partial correlations were computed using Spearman’s rank correlation, which does not assume linearity. Minor differences in case numbers reflect pairwise missing data. NAcc = nucleus accumbens; AQ = Autism Spectrum Quotient.

Bivariate correlation analyses revealed significant positive associations among the primary variables (Figure 1). Specifically, greater attentional switching impairment was associated with a larger right NAcc volume (r = 0.45, *p* = 0.012) and with increased nighttime screen time (r = 0.44, *p* = 0.014). A significant positive correlation was also observed between right NAcc volume and nighttime screen time (r = 0.455, *p* = 0.012), whereas no significant associations were found for the left NAcc. These relationships are shown in Figure 2. For exploratory purposes, we also examined correlations with total daytime screen time. No significant associations were observed between daytime screen time and NAcc volume or attentional switching (all *p* > 0.1). These associations also remained significant when ASD and ADHD diagnoses were included as covariates in partial correlation analyses.

A multiple regression was conducted to predict nighttime screen time from right NAcc volume and attentional switching impairment, with ASD and ADHD diagnoses entered as covariates. The overall model was statistically significant (F (2,44) = 3.92, *p* = 0.027) and accounted for 15.1% of the variance in nighttime screen time (R^2^ = 0.151). In this model, the right NAcc volume was a significant predictor of nighttime screen time (β = 0.33, *p* = 0.022), whereas attentional switching impairment did not significantly predict nighttime screen time (β = 0.18, *p* = 0.195). Full model details are provided in Table 2.

A post hoc mediation analysis was performed to test if attentional switching impairment mediated the relationship between right NAcc volume and nighttime screen time. The analysis did not yield a significant indirect effect (indirect effect = 0.004, 95% CI [−0.0002, 0.0113]). Thus, the mediation hypothesis was not supported (Supplementary Table S1).

Finally, the non-parametric sensitivity analysis on the subgroup of 28 participants with high data-logging adherence reinforced the primary findings. In this high-adherence group, nighttime screen time remained significantly correlated with both attentional switching impairment (ρ = 0.48, *p* = 0.030) and right NAcc volume (ρ = 0.58, *p* = 0.006). The association between right NAcc volume and attentional switching impairment showed a non-significant trend (ρ = 0.40, *p* = 0.075) (Supplementary Table S2).

## 4. Discussion

The present study investigated the neurostructural and cognitive correlates of problematic smartphone use (PSU), specifically examining the relationships between nucleus accumbens (NAcc) volume, attentional switching impairment, and nighttime screen time. Our results identified significant positive correlations between all three variables. However, when these factors were assessed simultaneously in a multiple regression model, only right NAcc volume remained a significant, independent predictor of nighttime screen time. This suggests the relationship between attentional switching and screen time may be accounted for by the underlying influence of NAcc structure. These findings were robust and persisted independently of comorbid diagnoses of Autism Spectrum Disorder (ASD) or Attention-Deficit/Hyperactivity Disorder (ADHD).

### 4.1. NAcc and Difficulty in Attention Switching

This study identified a significant positive correlation between NAcc volume and difficulty in attention switching among individuals with PSU. NAcc is a central node in the brain’s reward circuitry, mediating dopaminergic signaling involved in reward anticipation and motivational salience [21,22]. Notably, previous neurodevelopmental research has shown that structural overgrowth in subcortical regions such as the striatum, including the NAcc, is associated with reduced cognitive flexibility, particularly in populations with ASD [23]. These findings raise the possibility that the structural characteristics of the NAcc may be associated with difficulty in disengaging from ongoing activities, potentially through heightened sensitivity to reward-related cues and reduced flexibility in shifting attention. Although both left and right NAcc volumes were initially examined, only the right NAcc showed significant associations with attentional switching impairment in this study. Therefore, subsequent analyses focused on the right NAcc. Prior neuroimaging studies have also suggested hemispheric specialization of the ventral striatum, with right-lateralized connectivity linked to attentional control and cognitive flexibility [24]. Nevertheless, other studies have reported hemispheric asymmetries without consistent evidence for distinct functional roles [25].

### 4.2. Attentional Switching and Nighttime Screen Time

We also observed a positive correlation between difficulty in attention switching and nighttime screen time. However, this association did not remain significant in the multiple regression model once the right NAcc volume was included. This suggests that attentional switching difficulties may not be independently associated with nighttime screen use, but instead share variance with underlying neurostructural factors, with NAcc volume possibly representing a more robust correlate. Individuals with reduced attentional control may find it harder to disengage once they begin using their device, leading to extended screen time that continues beyond intended bedtimes [26]. This behavioral tendency may reduce total sleep duration and compromise sleep quality. Conversely, impaired attentional switching itself may promote bedtime procrastination and difficulty initiating sleep, as individuals remain engaged with stimulating content even when fatigued. Moreover, existing evidence indicates that sleep deprivation impairs executive function and self-regulation [18,27], potentially exacerbating pre-existing attentional control difficulties. These bidirectional effects suggest the presence of a self-reinforcing cycle in which impaired cognitive control and insufficient sleep contribute to and maintain maladaptive smartphone use patterns. This highlights the potential utility of interventions aimed at improving attentional flexibility and digital self-regulation, particularly for individuals with PSU.

### 4.3. NAcc and Nighttime Screen Time

In addition to its link with attentional switching, NAcc volume was also positively correlated with nighttime screen time. Prior research on behavioral and substance addictions has demonstrated that structural and functional abnormalities in the reward system—especially the striatum—can disrupt behavioral regulation and increase susceptibility to compulsive engagement [21,28]. Given the NAcc’s role in mediating reward-seeking and motivational behaviors, its association with prolonged smartphone use may reflect an underlying neurobiological predisposition toward heightened reward sensitivity, which could interfere with the capacity to regulate device use in the evening hours.

### 4.4. Limitations

Several limitations should be noted. First, the sample demonstrated a disproportionate representation of males and a high prevalence of ASD and ADHD comorbidity. Because participants were mainly children and adolescents brought to a specialized clinic by their families, the present findings may reflect only a subset of PSU cases, limiting generalizability to the wider population. Future community-based studies, including non-clinical and population-based samples, are warranted to confirm whether these neurocognitive associations generalize to broader and more diverse groups of smartphone users. Moreover, the study did not include a control group of non-clinical users, which restricts the interpretation of whether the observed correlations are specific to treatment-seeking individuals. Although smartphones were the primary devices used by participants, accurate tracking of other screen-based devices (e.g., tablets, PCs, game consoles) was not feasible, and thus their potential contribution could not be fully evaluated. Second, our measure of attentional switching relied solely on the AQ subscale, which is subjective, may overlap with ASD-related traits, and does not fully reflect actual executive functioning. Future studies should combine this with task-based assessments, such as the Wisconsin Card Sorting Test, to improve construct validity [29]. Third, our regression model accounted for only 15.1% of the variance in nighttime screen time, underscoring the multifactorial nature of PSU. Other variables—such as psychosocial context, sleep hygiene, or personality traits—likely play important roles but were not included in the present analysis [1]. A further limitation pertains to the completeness of the smartphone logging data. The median adherence rate was only 35.7% (equivalent to about 5 days of logs within the 14-day period), raising concerns about whether the recorded days accurately represented habitual use. Although our sensitivity analysis, restricted to participants with higher adherence, replicated the main findings, systematic bias cannot be ruled out. In our previous work, we found that logging adherence was positively correlated with treatment motivation [30], suggesting that lower adherence may reflect reduced motivation to engage in treatment. However, in the present sample, adherence rate was not significantly correlated with PSU severity, which provides some reassurance that missing data did not simply reflect more severe cases. It should also be noted that PSU severity and treatment motivation are not necessarily correlated, and low adherence may reflect motivational aspects rather than directly reflecting symptom severity. Finally, although nighttime smartphone use was measured, detailed sleep duration and quality were not the primary focus of this study. While sleep is an important confounding factor in state-dependent measures of attentional control, our measure was a trait-based questionnaire (AQ), making it less directly influenced by short-term fluctuations in sleep. Participants generally reported suboptimal sleep, but sleep indices did not show significant associations with NAcc volume, attentional switching, or nighttime screen time.

## 5. Conclusions

In conclusion, we identified a direct link between NAcc structure and nighttime screen time in individuals with PSU. Greater NAcc volume independently associated with more screen time and was also associated with impaired attentional switching. These results suggest that structural variations in this key reward-related region may underpin the difficulty in disengaging from rewarding digital content. By integrating measures of brain structure, cognitive control, and real-world behavior, this study provides a novel neurocognitive framework for understanding PSU.

## Figures and Tables

**Table 1:** Demographic, clinical, psychological, and neuroimaging characteristics of the participants (N = 53).

Characteristics	N = 53
Gender	
male	41 (77%)
female	12 (23%)
Age	
12–19	32 (60%)
20–29	19 (36%)
over 30	2 (4%)
Duration of illness (months)	24 (2–108)
ADHD	16 (30%)
ASD	18 (33%)
**Self-administered psychological scales**	**Median (min.–max.)**
SAS-SV (Smartphone Addiction Scale Short Version)	37 (15–52)
ADHD-RS (ADHD rating scale)	
ADHD-RS-Inattention subscale	12 (2–27)
ADHD-RS-Hyperactivity-Impulsivity subscale	4 (0–27)
AQ (Autism Spectrum Quotient)	21 (7–32)
AQ-Social Skills	5 (0–10)
AQ-Attention Switching	5 (2–10)
AQ-Attention to Detail	3 (0–9)
AQ-Communication	4 (0–8)
AQ-Imagination	3 (0–7)
**Log data and MRI data**	**Median (min.–max.)**
screen time (s)/day	15,491 (0–60,180)
nighttime screen time (s)/day*1	337 (0–20,527)
log acquisition rate*2	35.7 (0–100)
Left NAcc*3	0.459 (0.350–0.516)
Right NAcc*3	0.973 (0.810–1.085)

*1; Nighttime screen time(s)/day represents the amount of screen time between midnight and 6 a.m., divided by the number of days with available log data. *2; log acquisition rate is the ratio of the number of days on which logs were acquired to 14 days. *3; NAcc volume was normalized by dividing each participant’s NAcc volume by their total gray matter volume and then multiplying the result by the mean total gray matter volume across all participants.

**Table 2:** Multiple regression model to predict nighttime screen time.

Predictor Variables	Coefficient	Standardized Coefficient	t	*p*	VIF
Right NAcc	8825.523	0.330	2.371	0.022	1.005
Difficulty in attention switching	793.284	0.183	1.317	0.195	1.005
**Model Summary**					
**Observation**	**F (2,44)**	**Prob > F**	**R^2^**		
53	3.917	0.027	0.151		

Multiple regression predicting nighttime screen time (seconds per day, midnight–6 a.m.) from right nucleus accumbens (NAcc) volume (arbitrary units, normalized to total gray matter volume) and difficulty in attention switching (AQ subscale score, range 0–10). Diagnoses of ASD and ADHD were included as covariates. VIF = Variance Inflation Factor; AQ = Autism Spectrum Quotient.

## Data Availability

The data presented in this study are available on reasonable request from the corresponding author. The data are not publicly available due to privacy and ethical restrictions.

## Supplementary Materials

The following supporting information can be downloaded at: https://www.mdpi.com/article/10.3390/brainsci15101100/s1. Table S1: Summary of Mediation Analyses; Table S2: Spearman correlation coefficients among difficulty in attention switching, nighttime screen time, and right NAcc volume (n = 28).

## Abbreviations

The following abbreviations are used in this manuscript:

**Abbreviation**	**Definition**
ADHD	Attention-Deficit/Hyperactivity Disorder
ADHD-RS	Attention-Deficit/Hyperactivity Disorder Rating Scale
AQ	Autism Spectrum Quotient
ASD	Autism Spectrum Disorder
CAT12	Computational Anatomy Toolbox 12
CI	Confidence Interval
DSM-5	*Diagnostic and Statistical Manual of Mental Disorders*, Fifth Edition
IGD	Internet Gaming Disorder
KAKENHI	Grants-in-Aid for Scientific Research (Japan Society for the Promotion of Science)
MRI	Magnetic Resonance Imaging
NAcc	Nucleus Accumbens
PSU	Problematic Smartphone Use
SPM	Statistical Parametric Mapping
WCST	Wisconsin Card Sorting Test
