# Supplementary material for: Difficulty in Attention Switching and Its Neural Basis in Problematic Smartphone Use
Source: Brain Sci. 2025 Oct 13;15(10):1100. doi: 10.3390/brainsci15101100 (PMC12563531; doi:10.3390/brainsci15101100)
Supplement: Supplementary file 1 [file brainsci-15-01100-s001.zip › brainsci-3896910-supplementary.pdf]

Supplementary Materials:

Supplementary Table S1. Summary of Mediation Analyses

Model	X (IV)	M (Mediator)	Y (DV)	Total Effect (c)	Direct Effect (c′)	Indirect Effect (ab)	Boot 95% CI for ab
1	Difficulty in attention switching	nighttime screen time	NAcc Volume	0.0035 ($p = 0.6435$)	−0.0001 ($p = 0.9911$)	0.0036	[−0.0002, 0.0113]
2	NAcc Volume	difficulty in attentat switching	nighttime screen time	9964.03 ($p = 0.1666$)	8844.82 ($p = 0.1976$)	1119.21	[−1344.93, 15363.20]
3	nighttime screen time	difficulty in attentat switching	NAcc Volume	0.0000 ($p = 0.1666$)	0.0000 ($p = 0.1976$)	0.0000	[0.0000, 0.0000][1]

* c = total effect; c′ = direct effect; ab = indirect effect; CI = confidence interval. Indirect effect estimated using 5,000 bootstrap resamples via PROCESS macro (Model 4). Model 3 showed numerical instability.

https://doi.org/10.3390/xxxxx

Supplementary Table S2. Spearman correlation coefficients among difficulty in attention switching, nighttime screen time, and right NAcc volume (n = 28)

		**Spearman's ϱ**	**p-value**
**Difficulty in attention switching (AQ)**	nighttime screen time	0.475	0.030
**Difficulty in attention switching (AQ)**	Right NAcc	0.397	0.075
**nighttime screen time**	Right NAcc	0.575	0.006

*Correlations were computed using Spearman's rank-order correlation (two-tailed).
AQ = Autism Spectrum Quotient, Attention Switching subscale.
screen time = average screen time between midnight and 6 a.m.
Right NAcc volume = normalized right-side nucleus accumbens gray matter volume.
